# The Joint Effects of Social Norm Appeals and Fear Appeals in COVID-19 Vaccine Campaign Posters on Self-Perceived Communication Quality and Vaccination Intention

**DOI:** 10.3389/fpsyg.2022.760146

**Published:** 2022-03-28

**Authors:** Jiawei Liu, Xiaobing Yang, Yanqin Lu, Xia Zheng

**Affiliations:** ^1^School of Journalism and Communication, Jinan University, Guangzhou, China; ^2^School of Media and Communication, Bowling Green State University, Bowling Green, OH, United States; ^3^The Media School, Indiana University, Bloomington, IN, United States

**Keywords:** vaccine education, social norm appeal, fear appeal, communication quality, vaccination intention

## Abstract

To understand how different types of cues in vaccine education messages affect attitude toward campaign messages and vaccination intention, this study examined the impact of the presence of social norm appeals (individual vs. group cues) and the presence of fear appeals in coronavirus disease 2019 (COVID-19) vaccine campaign posters on perceived communication quality and vaccination intention. A 2 (social norm appeal: individual cue vs. group cue) × 2 (fear appeal: absence vs. presence) × 3 (repetition) within-subject factorial design experiment was conducted in China. Findings demonstrated that the presence of fear appeals in COVID-19 vaccine campaign posters elicited lower levels of perceived communication quality and vaccination intention than those without fear appeals. The interactive effect of fear appeals and social norm appeals was also found to be significant. Specifically, positive-framed messages (i.e., absence of fear appeals) with group cues and fear appeal messages with individual cues elicited higher perceived information quality and stronger vaccination intention than other types of messages. Understanding how these cues function jointly in COVID-19 vaccine campaign messages will help public health practitioners create more effective intervention strategies.

## Introduction

Vaccines are as important to our overall health as diet and exercise. According to the World Health Organization (2021), vaccines help develop immunity and prevent over 2 million deaths every year from diseases, such as diphtheria, tetanus, pertussis, influenza, and measles. After 2 years of living with coronavirus disease 2019 (COVID-19), the pandemic is still far from over and has caused nearly 5.55 million deaths across the world (John Hopkins University Center for System Science and Engineering, n.d.). Getting the COVID-19 vaccine helps reduce the risk of contracting coronavirus (Haynes, 2020). However, as of 19 January 2022, only a total of 3.92 billion people had been fully vaccinated, or 50.4% of the world’s population (John Hopkins University Center for System Science and Engineering, n.d.). Beneath the low COVID-19 vaccination rate lurks the vaccine hesitancy that has taken root in many countries (Dubé et al., 2015). For example, a subset of the population in different countries believe that vaccines harm the immune system and thus hesitate to vaccinate themselves and their children (Cooper et al., 2008; Omer et al., 2009; Edwards et al., 2016). Low vaccination rate and COVID-19 vaccine hesitancy can greatly decrease our ability to curtail the pandemic.

Reasons for COVID-19 vaccine hesitancy are complicated and encompass more than just a knowledge deficit. In China, vaccine hesitancy is also a growing public health problem. People were concerned about the side effects, safety, and lack of risk awareness (Sun, 2021). Current research has also confirmed that age, education, health literacy, rurality, and parental status affect COVID-19 vaccination intention among Chinese people (Wang et al., 2021; Zhang et al., 2021). For example, a study conducted by Wang et al. (2021) found that people from urban and suburban areas had lower vaccination willingness than those from rural areas. Zhang et al. (2021) surveyed *N* = 2,463 parents in China and found that parents of minor children (under age 18 years) were less likely to have their children get vaccinated against COVID-19. Meanwhile, it has been reported that older adults aged 70 and older were less willing to get vaccinated against COVID-19 (Liu, 2021).

Delivering vaccine education messages (e.g., COVID-19 vaccine campaign posters) may help boost vaccination intention. Current studies have found that the use of social norm appeals and fear appeals in vaccine education messages elicit greater vaccination intentions (e.g., Gerend and Shepherd, 2012; Iten et al., 2013; Lau et al., 2019) and higher levels of self-perceived message effectiveness (e.g., Kim et al., 2020). However, absent from the current literature is the interactive effect of fear appeals and social norm appeals. Our study seeks to fill this research gap using an online experiment. Thus, this study examined the main effects and the joint effects of social norm appeals and fear appeals on perceived communication quality and vaccination intention during COVID-19 vaccine campaign message possessing.

### Social Norm Appeal

The term “social norm” is defined as the self-perceived standards for what constitutes appropriate behavior that is based on widely shared beliefs about how individual members of a group ought to behave in a given situation (Elster, 1989). In other words, the presence of social norms in COVID-19 vaccine campaign messages may facilitate vaccination compliance intention thereby improving vaccination rates. Current research has found that health professionals, family members, and friends can play a significant role in adult vaccination uptake (e.g., Quinn et al., 2017; Elhadi et al., 2021; Graupensperger et al., 2021). For example, a survey study conducted by Quinn and her colleagues showed that high-risk populations were more likely to be vaccinated if they believed that most people around them wanted them to get vaccinated (Quinn et al., 2017). They also concluded that public health practitioners could reinforce positive social norms about the flu vaccine (Quinn et al., 2017). Graupensperger et al. (2021) had 647 undergraduate students complete an online survey, and their findings indicated that social norms regarding peers’ vaccination behaviors and attitudes were positively related to both the perceived importance of getting a COVID vaccine and vaccination intention. In addition, Elhadi et al. (2021) found that having a family member or friend infected with COVID-19 was positively correlated with the likelihood of vaccine acceptance. However, it is also important to note that they also found that having a family member or friend die due to COVID-19 was negatively correlated with the likelihood of vaccine acceptance.

Current research has also confirmed the influences of social norm appeals in health messages on vaccination intention and behavior (e.g., Gerend and Shepherd, 2012; Juraskova et al., 2012; Nyhan et al., 2012; Iten et al., 2013; Lau et al., 2019). In particular, some research studies explored the effects of different types of social norm cues in vaccine communication. For example, Lau et al. (2019) had participants randomly assigned to different conditions of a web-based experiment (including a control group and seven treatment groups with different vaccination coverage levels). Interestingly, their findings demonstrated that the presence of overall vaccination coverage (i.e., social norm appeal: group cue) did not always improve vaccination intention. Their findings suggest that the average vaccination intention was higher at lower coverage levels but lower at higher coverage levels. Another research study conducted by Iten et al. (2013) had vaccinated and unvaccinated healthcare workers at a Swiss hospital wore badges containing individual cues (“I am vaccinated against influence to protect you” vs. “I wear a mask to protect you”) during seasonal influenza epidemic to explain their vaccination choice to patients/visitors. The vaccination rate was significantly improved (to 37%) after a year. Thus, it seems that vaccination intention may vary depending on the presence of different types of social norm appeals (individual vs. group cues). It was not clear, however, whether exposure to different types of social norm cues in vaccine campaign messages alters how the health messages and the importance to get vaccinated are evaluated.

### Fear Appeal

The use of fear appeals to promote healthy behaviors is contentious (Avery and Park, 2018; Kim et al., 2020). A fear appeal is a persuasion technique that emphasizes the potential danger and harm that threaten the audience with negative, physical, psychological, and/or social consequences and motivate them to adopt the recommended behaviors (Hale and Dillard, 1995). For example, a typical fear appeal vaccine campaign message portrays negative consequences of vaccine hesitancy and refusal (such as getting sick with COVID-19 or even death). In the extended parallel process model (EPPM), Witte (1992) conceptualized fear appeal as the message depicting the components of threat (i.e., severity and susceptibility) and the components of efficacy (i.e., response efficacy and self-efficacy). Higher levels of perceived threat elicit fear and thereby activate the danger control process if perceived efficacy is also high (Witte, 1992). However, in the low-efficacy condition, fear arousal may result in defensive reactions, such as risk neglect or denial. Within the theoretical framework of EPPM, people are better motivated to get vaccinated against COVID-19 when both self-efficacy and perceived threat are high.

Previous studies have yielded mixed findings regarding the effect of fear appeals (e.g., So, 2013; Carcioppolo et al., 2017; Ort and Fahr, 2018; Kim et al., 2020; Su et al., 2021). For example, Kim et al. (2020) found that the presence of fear appeals was associated with greater motivation to process human papillomavirus (HPV) protection-related information. While some other research studies demonstrated that individuals self-reported a higher level of discomfort, less attention, and lower level of self-efficacy when seeing fear appeals in vaccine promotion messages (Ort and Fahr, 2018; Su et al., 2021). Thus, it is still unclear how fear appeals in vaccine campaign messages (e.g., the presence of negative pictures and content) affects motivations to control the danger or threat.

### Information Quality and Vaccination Intention

Effective information processing depends on communication quality and how the information is processed, among other things. Current research in the field of vaccine education has found that individuals prioritize information quality and are more likely to be vaccinated (Ghezzi et al., 2020; Di Gennaro et al., 2021; Su et al., 2021). Existing literature has also identified three major dimensions of information quality: the amount of information, believability, and interpretability (Lee et al., 2002).

The amount of information refers to “the degree to which the quantity or amount of available information is appropriate” (Kim et al., 2017, p. 694). While the amount of information should be sufficient enough for people to make informed decisions, too much information will cause cognitive overload and lead to information avoidance (Lee et al., 2002; Song et al., 2017). In this case, an appropriate amount of information conveyed in a persuasive message will help the formation of a positive attitude toward certain objects and issues (Baloglu and McCleary, 1999).

Believability refers to the extent to which information is considered true and credible (Wang and Strong, 1996). Information believability has been found to influence risk perceptions and behavioral changes in response to persuasive appeals, including advocacy for vaccination (Trumbo and McComas, 2003; Briñol and Petty, 2006). Fear appeals are likely to reduce information believability because individuals tend to avoid processing high-fear messages and thus consider them incredible (Dunbar et al., 2014).

Interpretability is defined as the extent to which information is explained with clear and unambiguous language (Wang and Strong, 1996). This dimension of information quality is particularly important in health communication messages because medical issues are often too complicated and technical to comprehend for a layperson (Salmon et al., 2021). An unclear message is likely to cause confusion and attitudinal ambivalence, which will lead to lower intentions to receive vaccines (Hofman et al., 2014; Kim et al., 2019).

COVID-19 vaccine campaign poster is a way of engaging target populations to get vaccinated. Therefore, it is important to examine how the use of fear appeals and social norm appeals in COVID-19 vaccine messages affects people’s perceived information quality and the subsequent vaccination intentions.

## Materials and Methods

### Experimental Design

A 2 (social norm appeal: individual cue vs. group cue) × 2 (fear appeal: absence vs. presence) × 3 (repetition) within-subject factorial design experiment was conducted in China. This design was fully crossed. Thus, participants viewed 12 COVID-19 vaccine campaign posters of four types: (1) COVID-19 vaccine campaign posters with both group cues and fear appeals, (2) COVID-19 vaccine campaign posters with both group cues only, (3) COVID-19 vaccine campaign posters with both individual cues and fear appeals, and (4) COVID-19 vaccine campaign posters with individual cues only. These posters were presented in a random order in this experiment.

### Participants

Participants (*N* = 859) who were living in China responded to the request to complete the online experiment. They were selected from multiple market research panels and got paid directly through Wenjuanxing (an alternative to Qualtrics in China). Participants’ age ranged from 18 to 65 years with an average age of 29.78 (*SD* = 7.27). Among these participants, 58.7% (*N* = 504) were women (refer to Table 1 for full details on demographic characteristics). Specifying a small effect size (0.15) and an α of 0.05 in the G*Power program (Faul et al., 2007), the proposed design requires at least 97 participants to have a 0.95 power estimate.

**TABLE 1:** Sample characteristics (*N* = 859).

	*M*(*SD*)	Percent
Age, year	30.02 (10.05)	
**Gender**		
Male	355	41.3
Female	504	58.7
**Education**		
High school grad or less	30	3.5
Occupational certificate or associate’s degree	92	10.7
Bachelor’s degree	672	78.2
Postgraduate degree	65	7.6
**Employment Status**		
Student	138	16.1
Employed	712	82.9
Unemployed	2	0.2
Retired	3	0.3
Others	4	0.5
**Individual income**		
Less than ¥1000/month	60	7
¥1001 – ¥5000/month	197	22.9
¥5001 – ¥10000/month	341	39.7
¥10001 – ¥20000/month	218	25.4
More than ¥20001/month	43	5
**Residence situation**		
Living alone	210	24.4
Living with parents	309	36.0
Living with roommate(s)	182	21.18
Others	158	18.4
**Fully vaccinated**		
Yes	691	80.4
No	168	19.6

### Stimuli

A total of 16 COVID-19 vaccine campaign posters presented in China were pretested to control for emotional arousal, positivity, and negativity to identify appropriate stimuli for this study. These posters were originally developed based on the objective criteria of social norm appeals (i.e., number of infected individuals) and fear appeals (absence vs. presence). Self-reported emotion of these 16 posters was collected from *N* = 35 undergraduate students who did not participate in the experimental session reported here. Totally, 12 out of 16 COVID-19 vaccine campaign posters were selected. Specifically, self-reported arousal [from 1 (low) to 7 (high)], positivity [from 1(low) to 7 (high)], and negativity [from 1 (strongly disagree) to 7 (strongly agree)] ratings were collected from 35 undergraduate students in the pretest. The results indicated that the 12 campaign posters selected for the final study did not elicit significant differences in self-reported emotional arousal [*F*(2,66) = 1.12, *p* = 0.331), negativity [*F*(2,66) = 2.36, *p* = 0.104], and positivity [*F*(2,66) = 2.54, *p* = 0.088] between posters within each cue category.

### Measures

#### Manipulated Independent Variables

##### Social Norm Appeal

This factor had two levels based on how many infected people were presented in the COVID-19 vaccine campaign posters: individual (= 1) vs. a group of people (≥ 2). Thus, COVID-19 vaccine campaign posters with individual cues contain only one person; while those with group cues include two or more persons. These social norm cues are assumed to be varied in the intensity of participation in collective action.

##### Fear Appeal

This factor had two levels: absence vs. presence. The fear appeal was manipulated by varying the presence of negative images and contents in COVID-19 vaccine campaign posters (e.g., unvaccinated people have a higher risk of dying from COVID-19).

##### Repetition

A total of 12 COVID-19 vaccine campaign posters were selected to represent the combination of manipulations as stated above. This was done to generalize ratings and responses to a type of ad circumstance rather than a specific poster. Health campaign messages are complex media messages that vary in a number of ways; by utilizing multiple exemplars of each cue type, we randomize extraneous features across conditions (Geiger and Newhagen, 1993).

#### Dependent Variable

##### Information Amount

Two items were adapted from a previous study (Lee et al., 2002) to assess information amount (e.g., “This information is of sufficient volume for our needs”). Items were rated on a five-point Likert-type scale, with responses ranging from 1 = *Strongly disagree* to 7 = *Strongly agree*. Higher scores indicated a greater level of self-perceived information amount (*M* = 4.67, *SD* = 1.11).

##### Information Believability

Two items from Lee et al.’s (2002) information quality assessment subscale were adapted to assess participants’ trust toward those selected COVID-19 vaccine campaign posters (e.g., “This information is believable”). Items were rated on a five-point Likert-type scale, with responses ranging from 1 = *Strongly disagree* to 7 = *Strongly agree*. Higher scores indicated a greater level of self-perceived information believability (*M* = 5.47, *SD* = 0.91).

##### Information Interpretability

Two items from Lee et al.’s (2002) information quality assessment subscale were adapted to measure information interpretability (e.g., “It is easy to interpret what this information means”). Items were rated on a five-point Likert-type scale, with responses ranging from 1 = *Strongly disagree* to 7 = *Strongly agree*. Higher scores indicated a greater level of self-perceived information interpretability (*M* = 4.77, *SD* = 1.16).

##### COVID-19 Vaccination Intention

A single item was adapted from a previous study (Ernsting et al., 2013) and used to assess participant’s vaccination intention after seeing each vaccine campaign poster (i.e., “If I haven’t got vaccinated yet, I would like to be vaccinated against COVID-19 within 3 months after seeing this message”). Item was rated on a seven-point Likert scale, with responses ranging from 1 = *Strongly disagree* to 7 = *Strongly agree*. Higher scores indicated a greater level of self-perceived COVID-19 vaccination intention (*M* = 6.1, *SD* = 1.08).

#### Analysis Strategy

Data were submitted to a 2 (social cue: individual eating, group eating) × 2 (fear appeal: absence, presence) × 3 (repetition) repeated-measures analysis of covariance (ANCOVA). The *p*-values and degrees of freedom corrected for sphericity assumption violation using the Greenhouse-Geisser method were reported, where appropriate. To control for the possibility that sociodemographic differences in the outcome variables might lead to spurious relationships, gender (1 = male and 2 = female), age, education (1 = high school grad or less, 2 = occupational certificate or associate’s degree, 3 = bachelor’s degree, and 4 = postgraduate degree) were entered as covariates in the repeated measures ANCOVA tests.

## Results

### Information Amount

**FIGURE 1:**
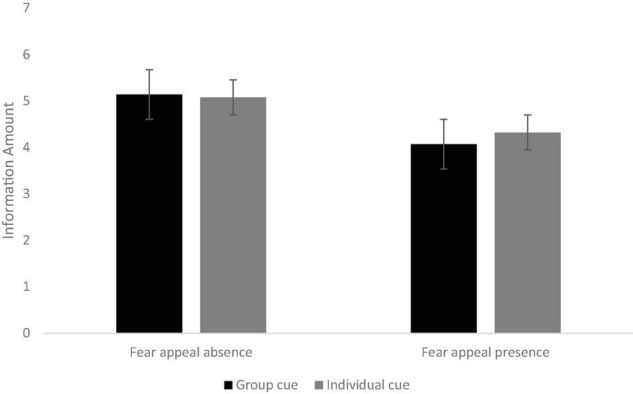
The interaction effect of social norm appeals (individual vs. group cues) and fear appeals (absence vs. presence) on information amount.

After controlling for age, gender, and education level, the interaction effect of social norm appeals and fear appeals was found to be significant: *F*(1,626) = 10.4648, *p* < 0.01, η*_*p*_^2^* = 0.02. As can be seen from Figure 1, posters with group cues (*M* = 5.14, *SD* = 0.053) and individual cues (*M* = 5.08, *SD* = 0.06) had significantly higher ratings of information amount compared with those with both social cues and fear appeals (posters with both group cues and fear appeals: *M* = 4.07, *SD* = 0.08, *p* < 0.001; posters with both individual cues and fear appeals: *M* = 4.32, *SD* = 0.08, *p* < 0.001). COVID-19 vaccine campaign posters with individual cues and fear appeals had significantly higher ratings of information amount (*M* = 4.332, *SD* = 0.051) than those with both group cues and fear appeals (*M* = 4.08, *SD* = 0.05, *p* < 0.001). A significant main effect of fear appeals was found on information amount: *F*(1,626) = 62.7, *p* < 0.001, η*_*p*_^2^* = 0.09. Posters without fear appeals (*M* = 5.11, *SD* = 0.05) had significantly higher ratings of information amount than those with fear appeals (*M* = 4.2, *SD* = 0.08, *p* < 0.001). However, no significant differences were found between posters with group cues and individual cues (*F* < 1, *p* = 0.99).

### Information Believability

**FIGURE 2:**
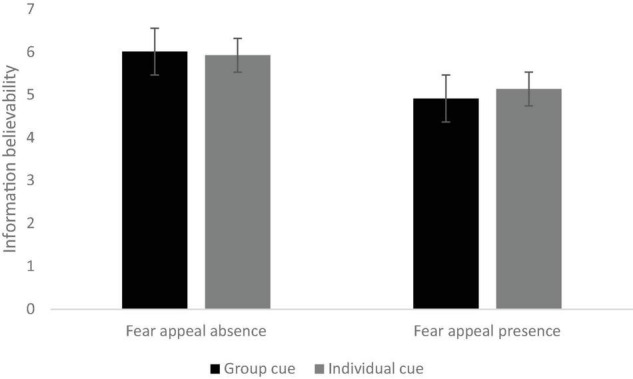
The interaction effect of social norm appeals (individual vs. group cues) and fear appeals (absence vs. presence) on information believability.

After controlling for age, gender, and education level, the interaction effect of social norm appeals and fear appeals was found to be significant: *F*(1,626) = 12.92, *p* < 0.001, η*_*p*_^2^* = 0.02. As can be seen in Figure 2, posters with group cues had the highest ratings of information believability (*M* = 6.01, *SD* = 0.04, *p* < 0.05). Meanwhile, posters with group cues and fear appeals had the least ratings of information believability (*M* = 4.92, *SD* = 0.07, *p* < 0.001). A significant main effect of fear appeals was found on information believability: *F*(1,626) = 43.39, *p* < 0.001, η*_*p*_^2^* = 0.07. Posters without fear appeals (*M* = 5.97, *SD* = 0.07) had significantly higher ratings of information believability than those with fear appeals (*M* = 5.03, *SD* = 0.04, *p* < 0.001). However, no significant differences were found between posters with group cues and individual cues (*F* < 1, *p* = 0.35).

### Information Interpretability

**FIGURE 3:**
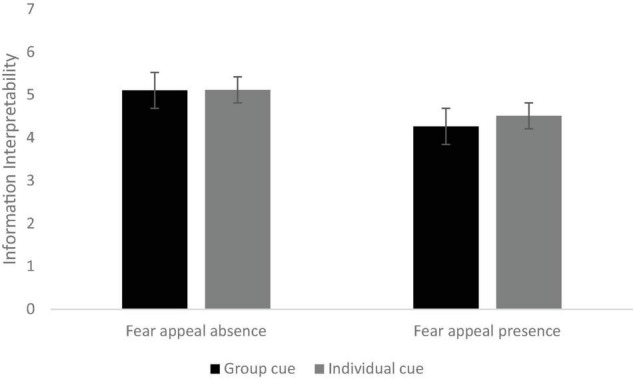
The interaction effect of social norm appeals (individual vs. group cues) and fear appeals (absence vs. presence) on information interpretability.

After controlling for age, gender, and education level, the interaction effect of social norm appeals and fear appeals was found to be significant: *F*(1,626) = 6.23, *p* < 0.05, η*_*p*_^2^* = 0.01. As can be seen from Figure 3, posters with group cues (*M* = 5.11, *SD* = 0.06) and individual cues (*M* = 5.12, *SD* = 0.06) had significantly higher ratings of information interpretability compared with those with both social cues and fear appeals (posters with both group cues and fear appeals: *M* = 4.27, *SD* = 0.08, *p* < 0.001; posters with both individual cues and fear appeals: *M* = 4.51, *SD* = 0.08, *p* < 0.001). COVID-19 vaccine campaign posters with individual cues and fear appeals had significantly higher ratings of information interpretability (*M* = 4.51, *SD* = 0.08) than those with both group cues and fear appeals (*M* = 4.27, *SD* = 0.08, *p* < 0.001). A significant main effect of fear appeals was found on information interpretability: *F*(1,626) = 38.94, *p* < 0.001, η*_*p*_^2^* = 0.06. Posters without fear appeals (*M* = 5.11, *SD* = 0.05) had significantly higher ratings of information interpretability than those with fear appeals (*M* = 4.39, *SD* = 0.08, *p* < 0.001). However, no significant differences were found between posters with group cues and individual cues (*F* < 1, *p* = 0.5).

### COVID-19 Vaccination Intention

After controlling for age, gender, and education level, the main effect of fear appeals was found to be significant: *F*(1,626) = 20.3, *p* < 0.001, η*_*p*_^2^* = 0.03. Posters without fear appeals (*M* = 5.11, *SD* = 0.05) had significantly higher ratings of COVID-19 vaccination intention than those with fear appeals (*M* = 4.39, *SD* = 0.08, *p* < 0.001). However, the interaction effect of social norm appeals and fear appeals (*F* < 1, *p* = 0.91) and the main effect of social norm appeals (*F* < 1, *p* = 0.53) were found to be insignificant.

## Discussion

The goal of this study was to investigate the joint effect of social norm appeals (individual vs. group cues) and fear appeals (absence vs. presence) in promoting COVID-19 vaccination. In general, we found that the use of fear appeals would not help increase (or may even discourage) participants’ perceived information quality and the subsequent vaccination intentions. Participants self-reported significantly lower levels of information amount, information believability, information interpretability, and COVID-19 vaccination intention after exposure to the posters with fear appeal than those without fear appeals. Our results are consistent with previous studies (Ort and Fahr, 2018; Su et al., 2021). The Chinese government has been using strict control measures (e.g., travel restrictions and a 14-day quarantine strategy for international travelers) to fight against COVID-19 since the pandemic began. Public fear of COVID-19 gets low due to the dramatic decline in COVID-19 cases in China. In this case, giving clear instruction to the general public about why it is necessary to get vaccinated may be more effective than scaring them. Thus, it is important to omit fear appeals to avoid developing counter-productive vaccination campaign messages.

In addition to the main effect of fear appeals, this study also found that fear appeals interact with social norm appeals in affecting perceived information quality and vaccination intentions. On the one hand, the presence of group cues elicited greater self-perceived information quality and vaccination intentions during exposure to positive-framed messages than exposure to fear appeal messages; on the other hand, the presence of individual cues elicited greater self-perceived information quality and stronger vaccination intentions during exposure to fear appeal messages than exposure to positive-framed messages. Consistent with previous studies on the promotion of other vaccines (e.g., Gerend and Shepherd, 2012; Juraskova et al., 2012; Nyhan et al., 2012; Iten et al., 2013; Lau et al., 2019), these findings suggest that *the use of group cues in positive-framed messages* and *the use of individual cues in fear appeal messages* would be effective strategies in the design of COVID-19 promotion materials in China. As suggested by classic economic theories of decision-making (Simon, 1959), people are better motivated to make changes for their own benefit and they often care more about their own welfare under threatening situations. In this case, it is conceivable that fear appeal health messages that emphasize self-interest in COVID-19 vaccination actions would trigger stronger defensive responses (e.g., getting vaccinated) than messages that emphasize cooperative efforts. Instead of emphasizing “we will get infected without vaccination,” stressing “I will get infected without vaccination” could better address vaccine hesitancy and motivate vaccination intention. Thus, public health professionals should consider the joint effects of fear appeals and social norm appeals when developing vaccination campaign messages that resonate effectively with target audiences.

Limitations of this study include issues regarding the stimulus and experimental controls. First, the stimuli were presented in an online experiment in which the messages appear as screenshots as opposed to printed posters at public places. This may limit the external validity, although it allowed us to have more control over exposure than other methods to examine the interactive effect of social norm appeals and fear appeals. In addition, we pretested the selected poster stimuli and used a multiple message design [see Geiger and Reeves (1993)] in this study to randomly spread message variance caused by other factors across cells and maximize control of message heterogeneity (Slater et al., 2015). It is possible that confounds may still exist. Thus, these findings should be replicated using other messages in future studies. Finally, this study was conducted among Chinese in Mainland China. It would be interesting to test the messages with other populations, especially those with different cultural backgrounds or those who are at higher risk of getting COVID-19 might perceive the messages differently. Since there are limited studies on investigating the interactive effects of social norm appeals and fear appeals, more research is needed about the joint influences of social norm appeals and fear appeals on individual emotional, cognitive, and behavioral responses.

Taken together, the results of this study have important implications for future research and vaccine promotion in many ways. Our findings suggest that the presence of fear appeals in COVID-19 vaccine campaign messages may not help motivate individuals to get vaccinated. Furthermore, we identified the joint effects of fear appeals and social norm appeals. Both positive-framed messages (no fear appeals) with group cues and fear appeal messages with individual cues elicit greater self-perceived information quality and vaccination intentions (compared with positive framed messages with individual cues and fear appeal messages with group cues). Practically, the findings should provide researchers and public health practitioners with important insights into the design of COVID-19 vaccine campaign messages. Changing the way the COVID-19 vaccination is promoted could improve the uptake of the COVID-19 vaccines, thereby getting coverage rates higher.

## Data Availability Statement

The raw data supporting the conclusions of this article will be made available by the authors, without undue reservation.

## Ethics Statement

The studies involving human participants were reviewed and approved by the Jinan University Institutional Review Board. Written informed consent for participation was not required for this study in accordance with the national legislation and the institutional requirements.

## Author Contributions

JL contributed to conceptualization, questionnaire design, data collection, data analysis, and manuscript writing. XY contributed to conceptualization, questionnaire design, data collection, and editing. YL wrote and edited the manuscript. XZ wrote the manuscript. All authors contributed to the article and approved the submitted version.

## Conflict of Interest

The authors declare that the research was conducted in the absence of any commercial or financial relationships that could be construed as a potential conflict of interest.

## Publisher’s Note

All claims expressed in this article are solely those of the authors and do not necessarily represent those of their affiliated organizations, or those of the publisher, the editors and the reviewers. Any product that may be evaluated in this article, or claim that may be made by its manufacturer, is not guaranteed or endorsed by the publisher.
